# Elevated Temperature Performance of Reactive Powder Concrete Containing Recycled Fine Aggregates

**DOI:** 10.3390/ma13173748

**Published:** 2020-08-24

**Authors:** Hammad Salahuddin, Liaqat Ali Qureshi, Adnan Nawaz, Muhammad Abid, Rayed Alyousef, Hisham Alabduljabbar, Fahid Aslam, Summera Fahmi Khan, Rana Faisal Tufail

**Affiliations:** 1 Civil Engineering Department, COMSATS University Islamabad, Wah Campus, Wah Cantt 47040, Pakistan; hammad.salahuddin@ciitwah.edu.pk (H.S.); adnan.nawaz@ciitwah.edu.pk (A.N.); summera.fahmi@ciitwah.edu.pk (S.F.K.); faisal.tufail@ciitwah.edu.pk (R.F.T.); 2 Civil Engineering Department, University of Engineering and Technology, Taxila 47050, Pakistan; 3 Civil Engineering Department, Swedish College of Engineering and Technology, Wah Cantt 47040, Pakistan; liaqat.qureshi@scetwah.edu.pk; 4 College of Aerospace and Civil Engineering, Harbin Engineering University, Harbin 150001, China; 5 Department of Civil Engineering, College of Engineering in Al-Kharj, Prince Sattam bin Abdulaziz University, Al-Kharj 11942, Saudi Arabia; r.alyousef@psau.edu.sa (R.A.); h.alabduljabbar@psau.edu.sa (H.A.); f.aslam@psau.edu.sa (F.A.)

**Keywords:** reactive powder concrete (RPC), C & D waste management, recycled fine aggregates, recycled aggregate concrete, elevated temperature

## Abstract

This study examines the effect of elevated temperature on various properties of reactive powder concrete (RPC) containing varying percentages of recycled fine aggregates as sand replacement. Recycled fine aggregates were collected from two sources, i.e., demolished normal strength concrete and demolished RPC. The specimens were prepared using 25%, 50%, and 75% replacement of natural sand with recycled fine aggregates, exposed to two different curing conditions and were subjected to four temperatures, i.e., 25, 200, 400, and 600 °C. Later, the specimens were tested for mass loss, compressive strength test, split-tensile strength test, flexural strength test, and water absorption test at all temperature ranges. Results determined that although the mechanical properties degraded with the temperature rise, the recycled aggregates can be employed as a partial replacement of natural sand in RPC without causing a significant decrease in the performance of RPC, and can help to produce more sustainable RPC by using recycled aggregates.

## 1. Introduction

With the increasing growth in urbanization, the construction industry has witnessed an enormous increase in the consumption of construction materials including concrete. This has led to the generation of an increased amount of Construction and Demolition (C &D) waste, which has caused negative impacts on the environment. C &D waste mainly comes from concrete, bricks, ceramic, glass, etc. Crushing and sorting of concrete waste results in recycled coarse aggregates (RCA) and recycled fine aggregates (RFA). Management of C &D waste has always been a challenging task and the use of RCA and RFA is considered as one of the promising solutions to this problem [1].

Recent studies have focused on many new types of high-strength concrete materials, including reactive powder concrete (RPC), which is famous due to its superior mechanical and durability properties and very low permeability. The main reasons for the significant improvement in the performance of RPC are the removal of coarse aggregates to eliminate the weakest link in the matrix, dense microstructure due to addition of pozzolanic materials [2], very low water to binder ratio, i.e., as low as 0.13 [3], particle gradation that results in lesser porosity [4], improved curing methods [5], and the use of micro and/macro steel fibers [6,7]. Its compressive strength ranges from 150–800 MPa, fracture energy in the range of 1200–40,000 J/m^2^, and an ultimate tensile strain at the order of 1% [8,9]. The use of hybrid steel fibers, i.e., straight and hooked, has been reported to improve the flexural and tensile strength of RPC in contrast to when only micro steel fibers were employed [10,11,12]. The use of waste steel fibers has also proved to be effective and sustainable for the production of RPC [13,14]. On one hand, increase in cement content and removal of cheaper coarse aggregates has increased the cost of RPC, yet, on the other hand, it has offered benefits of reducing members’ cross-section that leads to reduced dead load [15], and partial or total removal of passive reinforcement due to the use of fibers [16].

The literature survey expresses that there have been many studies that have utilized different pozzolanic materials like silica-fume [17], fly-ash, ground granulated blast furnace slag, and other waste materials as a partial replacement of cement to produce sustainable concrete with a better microstructure and improved performance [18,19,20,21,22]. Moreover, the use of different waste materials, including the C & D waste as a coarse aggregate replacement [23,24,25,26,27,28,29,30,31], and as a substitute for fine aggregates in the conventional concrete [32,33,34,35,36,37,38,39] as well as in RPC [40], has also been reported. Sand obtained from river mining is one of the main elements of RPC, which is used as a filler [41]. Rapid development in the construction industry has posed a risk to the sand reserves due to excessive and unplanned mining. River sand takes thousands of years to form but its current consumption rate is much higher than its restoration rate. To safeguard from these negative impacts of river mining on navigation, flood control, and river ecology [42], it has become necessary to look for alternative materials as a replacement of natural sand. These measures can help to address the problem of solid waste management and can also reduce the material cost of RPC. One of the solutions to address this problem is to use RFA as a substitute for natural river sand in RPC. Although RCA has been successfully utilized in the conventional concrete and is being incorporated in several applications of the construction sector, the use of RFA is very limited due to its poor physical properties including lower hardness, lower density, higher water absorption capacity due to a large amount of adhered mortar, and lower strength properties as compared to the natural sand [43].

The effect of RFA in the conventional concrete at room temperature has been studied by several researchers and it was found that RFA causes a decrease in the compressive strength, split-tensile strength, and elastic modulus of concrete mixes [33,34,35,36,37]. Several other researchers reported an improvement in the performance of conventional concrete after replacing natural fine aggregates with RFA [44,45]. Few studies have been carried out on the behavior of UHPC containing RFA. Zhang et al. [46] reported a negative effect of RFA on the microstructure and mechanical properties of UHPC. Yu et al. [47] and Salahuddin et al. [40] found that RFA improves the performance of UHPC/RPC if used in an optimum amount.

The risk of fire or exposure to elevated temperatures is one of the common hazards faced by structures throughout their life span. This hazard has attracted the attention of researchers to study the elevated temperature performance of concrete made with recycled aggregates to avoid structural damage and loss of lives in the case of a fire incident. In the recent past, many researchers have studied the behavior of RPC at elevated temperatures [15,48,49,50,51,52,53,54,55,56,57,58,59] and determined that RPC is susceptible to explosive spalling when exposed to elevated temperatures due to thermal stress induced by rapid temperature rise and water vapor, which may cause high pore vapor pressure. The use of steel fibers, polypropylene (PP) fibers, and the combination of steel and PP fibers is helpful to prevent explosive spalling [49,50,59]. Melting of PP fibers at around 167 °C creates micro-channels, which helps the trapped vapors to release, thus decreasing the vapor pressure [59]. The use of PP fibers is also helpful in reducing the drying shrinkage and crack width in the cement matrix due to their widespread dispersion in the concrete mix that makes the concrete more durable [60,61]. Additionally, the use of steel fibers enhances the resistance of fire-induced spalling by increasing the tensile capacity of concrete [58]. Thus, the incorporation of hybrid steel and PP fibers enhances the performance of RPC under elevated temperatures without the risk of explosive spalling.

Although several studies have been conducted to study the performance of conventional and high strength concrete containing RFA, as per the authors’ knowledge, no research has been performed until now to study the elevated temperature performance of RPC containing RFA as a sand replacement. In this study, RPC was prepared using two different types of RFA, i.e., first one acquired by recycling the conventional concrete and termed here as RFA-NSC, and the other one obtained from demolished RPC and termed here as RFA-RPC. Both types of RFA were added separately as a substitution of 25%, 50%, and 75% of natural sand. The prepared samples were then subjected to two curing conditions (normal and hot water curing) before exposure to four temperature ranges, i.e., 25, 200, 400, and 600 °C. The exposed samples were then tested for mass loss, compressive strength, split-tensile strength, flexural strength, and water absorption tests. Stress-strain curves were also generated for all mixes and equations were derived to predict various parameters. The effect of two different dosages of PP fiber was also examined at 25% replacement of sand with RFA.

## 2. Materials and Methods

### 2.1. Materials

**Figure 1:**
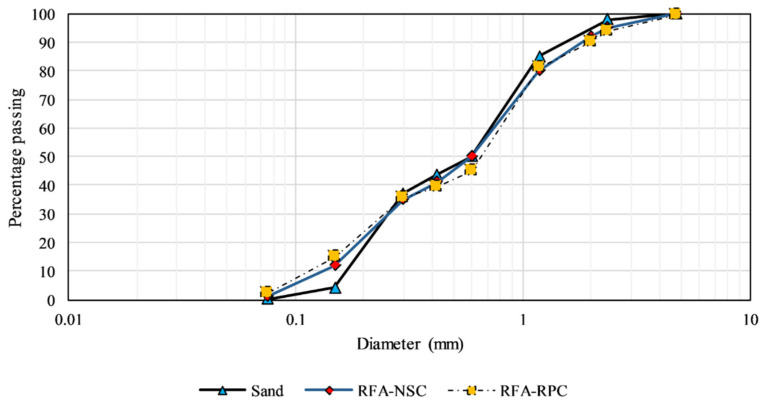
Grading curve of fine aggregates.

The cementitious materials employed in this study were Ordinary Portland Cement (OPC) conforming to ASTM C150 [62] and silica fume, in compliance with ASTM C1250 [63]. The chemical and physical properties of both the binders were provided by the manufacturer and are listed in Table 1 and Table 2, respectively. The physical properties of OPC, including the specific surface area, specific gravity and setting time of OPC were also confirmed as per ASTM C204-18 [64], ASTM C188-17 [65] and ASTM C191-19 [66], respectively, whereas ASTM C136/C136M-14 [67], ASTM C29/C29M-17 [68], and ASTM C128-15 [69] were employed to determine the physical properties of aggregates, as listed in Table 3. To cut down the water demand and to make the mix workable, an ultra-high range water reducing agent was used. Grey sand, collected from a riverbed quarry and in compliance with ASTM C778 [70], was added as a filler. Varying percentages of recycled fine aggregates (RFA), obtained from i) recycled normal strength concrete (RFA-NSC), and ii) Recycled reactive powder concrete (RFA-RPC), were added as a partial substitute of natural river sand. RFA-NSC was prepared by demolishing concrete blocks available at a local source, whereas RFA-RPC was acquired from the lab prepared RPC specimens, which exhibited compressive strength of 130–140 MPa at the age of 28 days. Before using as a sand replacement, a crusher plant was employed to crush the recycled materials to smaller pieces, which were further reduced to an appropriate size to be added as fine aggregates. Figure 1 illustrates the particle size gradation of the fine aggregates incorporated in this study, along with Table 3 presenting the physical properties of these materials. Hybrid fibers were used in all specimens, i.e., copper-coated straight steel fibers, hooked steel fibers, and polypropylene (PP) fibers. PP fibers were used to prevent the explosive spalling at elevated temperature [59,71]. To study the dosage effect of PP fibers, samples with 25% sand replacement were prepared by adding 0.5% and 1% of PP fibers by volume of concrete. For the remaining samples, the dosage of PP fibers was kept constant at 0.5% by concrete volume. The physical properties of fibers were provided by the manufacturer and are listed in Table 4.

### 2.2. Mix Proportion

Table 5 presents the mix proportions used in this study. For convenience, all mixes were assigned an ID to specify the replacement percentage and type of the recycled material used. A total of nine mixes were prepared. The control mix was designated as C, and N-25, N-50, and N-75 represent the mixes containing RFA-NSC, replacing natural sand by 25%, 50%, and 75%, respectively. H-25, H-50, and H-75 represent the mixes containing RFA-RPC, replacing natural sand by 25%, 50%, and 75%, respectively. At 25% sand replacement ratio, for both N-25 and H-25, the dosage of PP fibers was varied from 0.5% to 1% of RPC volume (designated as N-25-1P and H-25-1P) to study the effect of PP fibers dosage. For all other mixes, PP fibers content was kept constant at 0.5% of RPC volume. Water to binder ratio of 0.21 was used for all mixes. Moreover, the amount of superplasticizer was kept at 2% by weight of the binder. A ratio of 1:1.1:0.25 (cement: sand: SF) was observed for the control mixture.

### 2.3. Specimen Preparation

The planetary mixer was used to prepare the RPC mixes. First of all, silica-fume and cement were mixed in the dry state for 2 min. Then 80% water along with half of SP, was added and mixed for 5–6 min to attain uniformity of mixture. At that point, RFA was added to the mixer. After 2–3 min of mixing, the remaining water and SP were then introduced to the mixture. After mixing for 2–3 min, the natural river sand was introduced in the mixer and was further mixed for 3–4 min. After the mixture reached fluid-like state, steel fibers and PP fibers were added and mixed for further 2–3 min to ensure uniform dispersion of fibers. The total mixing time for the preparation of the RPC mix was around 20 min. The flow test of the RPC mix was conducted as per ASTM C1437 [72].

After casting into molds, the specimens were covered with plastic sheets and demolded after 24 h. The specimens of all RPC mixes were divided into two groups. The first group was subjected to normal water curing (NC) at room temperature, whereas the other group was initially exposed to hot water curing (HC) at 90 °C for 48 h, followed by immersion in water at room temperature until the age of testing.

## 3. Experimental Program

After 28 days of curing, the samples were withdrawn from the curing tank and oven-dried for 24 h to prevent spalling of concrete [73], before exposure to the elevated temperatures. The samples were then subjected to four temperatures (25, 200, 400, and 600 °C) after measuring the initial mass of the samples. For elevated temperature exposure, a muffle furnace was used with a heating rate of 5 °C per min, until the required temperature was attained. The samples were then kept at the target temperature for about two hours to ensure the homogeneity of temperature. After exposure to each target temperature, the samples were cooled down to room temperature and kept for 24 h before testing. The compressive strength test was conducted following ASTM C109 [74] on cube specimens of 50 mm × 50 mm × 50 mm using displacement controlled universal testing machine. The split-tensile strength test was conducted as per ASTM C496 [75] on 100 mm × 200 mm cylindrical specimens. Flexural strength test was conducted following ASTM C293 [76] while the water absorption test was conducted following ASTM C642 [77]. The summary of test methods and specimen details is given in Table 6.

## 4. Results

### 4.1. Flow Test

**Figure 2:**
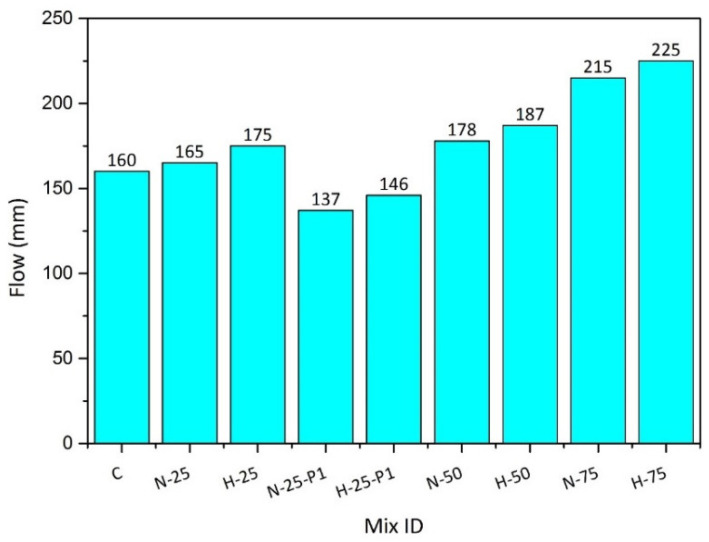
Comparison of the flow of tested RPC mixes.

The results of the flow test, measured in mm, are presented in Figure 2. It was observed that the use of ultra-high range water reducer made it possible to achieve the flow of all RPC mixes ranging between 160 mm and 225 mm. The values of flow tend to increase as the content of both RFAs increased. The reason for the increased degree of flow was the supplementary water, which was provided to compensate for the water demand of recycled aggregates whose water absorption was higher than that of the natural fine aggregates. The mixes containing RFA-RPC aggregates experienced higher flow than the mixtures containing RFA-NSC, as the water absorption of RFA-RPC was higher than that of RFA-NSC and, hence, more water was required for the RPC mixes containing RFA as a sand replacement. Moreover, mixes with 1% PP fibers exhibited lower flow values as compared to the mixes with 0.5% PP fibers. This reduction in flow was due to the hindrance caused by the high volume of PP fibers, which caused resistance in the flow of the RPC mix.

### 4.2. Mass Loss

**Figure 3:**
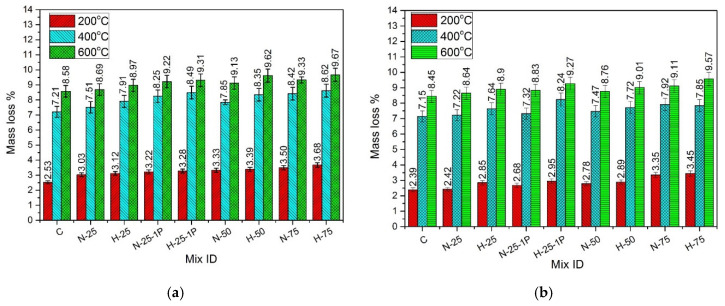
Mass loss of specimens cured under (**a**) normal curing, and (**b**) hot water curing.

Mass of all samples was determined before and after the heat treatment, using a scale with 0.1 g accuracy. The difference between the initial and final mass was reported as percentage mass loss. Figure 3 illustrates the percentage mass loss for all specimens containing varying content of both types of RFAs, exposed to normal and hot water curing conditions. The results show that at all exposure temperatures, the mass loss increased as the RFA content increased from 0% to 75%. This was due to the higher water absorption capacity of RFA as compared to the natural sand due to which the initial water content of samples with higher RFA content was higher than those containing lower RFA content. Moreover, for all specimens, the mass loss increased with an increase in temperature and the trend was almost similar for both the normal cured and hot water cured specimens. The percentage mass loss at 200 °C was in the range of 2.53–3.68% for the specimens under normal water curing and it was in the range of 2.39–3.45% for the specimens under hot water curing. Overall, at any temperature, the hot cured specimens exhibited slightly lower mass loss as compared to the normal cured specimens. The mass loss at 200 °C was due to the melting of PP fibers, with a melting point of 160 °C, which allowed the release of bound water from the matrix [78]. The mass loss at and beyond 400 °C was more significant than it was at 200 °C. This rapid increase in mass loss was attributed to the decomposition of C-S-H and Ca(OH)_2_ [79,80]. At any exposed temperature, the mass loss of specimens containing RFA-RPC was slightly higher than those containing RFA-NSC. This was due to the higher water absorption of RFA-RPC because of which greater initial water content was present in the mixes containing RFA-RPC, which consequently led to higher mass loss as compared to the mixes containing RFA-NSC. The mass loss also increased with the increase of PP fibers from 0.5% to 1% because greater number of PP fibers available in the mix resulted in higher number of molten fibers at elevated temperature [71] and, thus, higher mass loss was observed in the case of samples with 1% PP fibers.

### 4.3. Stress-Strain Curves

**Figure 4:**
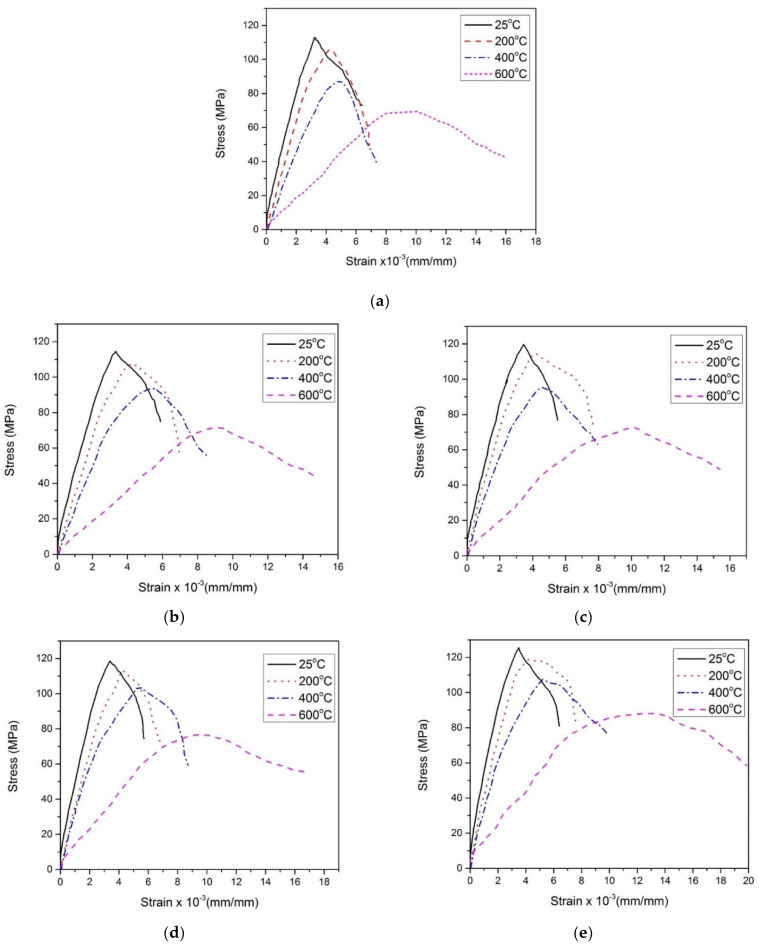

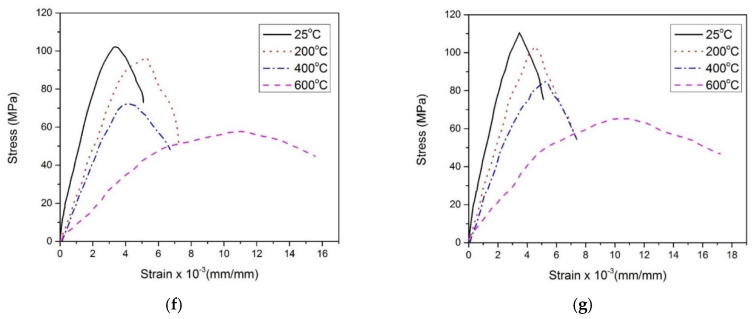
Stress-strain response of RPC specimens under normal curing conditions, (**a**) C, (**b**) N-25, (**c**) H-25, (**d**) N-50 (**e**) H-50, (**f**) N-75, (**g**) H-75.

**Figure 5:**
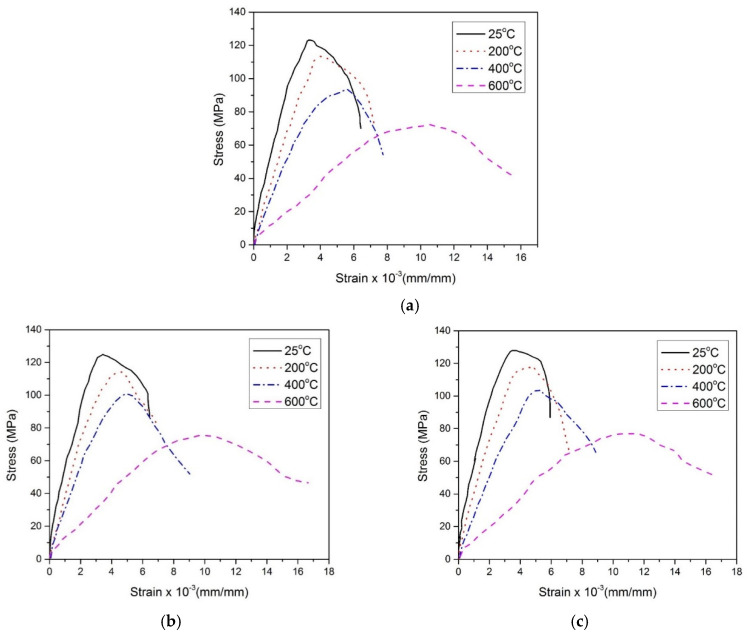

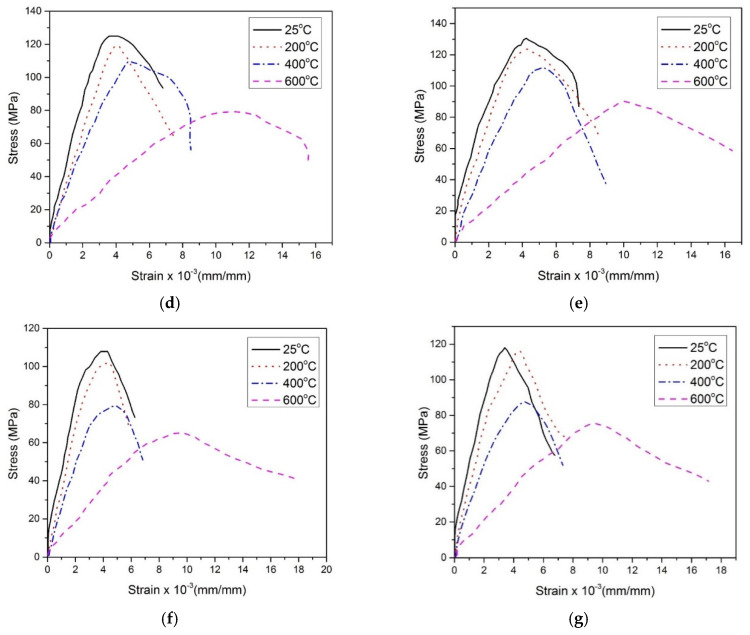
Stress-strain response of RPC specimens under hot curing conditions, (**a**) C, (**b**) N-25, (**c**) H-25, (**d**) N-50 (**e**) H-50, (**f**) N-75, (**g**) H-75.

The compressive stress-strain behavior of all mixes containing RFA-NSC and RFA-RPC, at various temperature exposures and under both normal and hot curing conditions, is shown in Figure 4 and Figure 5, respectively. A displacement-controlled test setup was used to capture the complete behavior including ascending and descending branches of the stress-strain curve. All samples exhibited ductile behavior that was evident from the descending branch of the curves. As reported in the previous studies, it was due to the addition of hybrid fibers that prevent the breaking of specimens into small pieces by prohibiting crack opening and propagation. Hooked fibers prevented the pullout of concrete while micro straight fibers bridged the gap and prevented crack opening and propagation [10,11,12]. All RPC mixes showed similar behavior at all temperatures, i.e., as the samples were exposed to the elevated temperatures of 200 and 400 °C, there was no significant change in the shape of the curve except that the slope of the curves started decreasing and peak strain started increasing along with a decrease in compressive strength. For all mixes, the shape of curves flattened when the samples were exposed to 600 °C with an increase in peak strain and a decrease in elastic modulus and compressive strength. This can be attributed to changes in microstructure at elevated temperature, i.e., development of cracks in the mortar, weakening of bond due to thermal incompatibility of paste-aggregate, decomposition of CH, and disintegration of CSH gel [81]. There was no significant effect of two different curing conditions on the shape of the curves. The effect of hot curing condition was reflected in the improvement of various parameters of the curve, such as peak compressive stress, peak strain, and elastic modulus, and are discussed in detail in the relevant sections.

### 4.4. Compressive Strength

**Figure 6:**
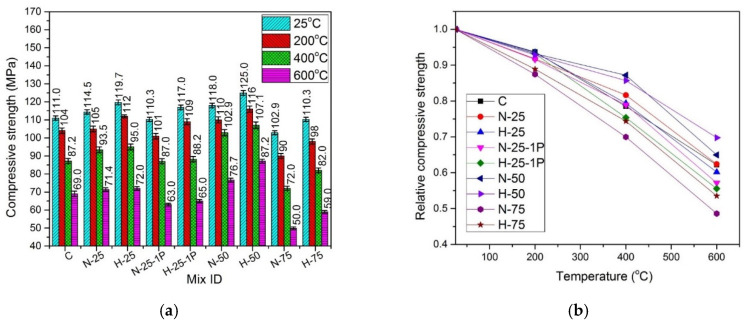
Comparison of compressive strength of specimens cured under normal conditions (**a**) absolute, (**b**) relative.

**Figure 7:**
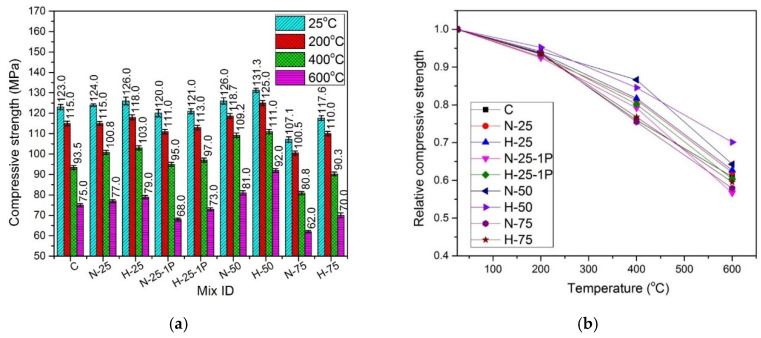
Comparison of compressive strength of specimens cured under hot water conditions: (**a**) absolute, and (**b**) relative.

The absolute and relative compressive strength values for all mixes cured under normal and hot water conditions are presented in Figure 6 and Figure 7, respectively. The relative compressive strength values were obtained by dividing the residual strength at an elevated temperature with the original strength at 25 °C.

#### 4.4.1. Effect of Recycled Aggregate Content and Type

Under both curing conditions and for both RFA-NSC and RFA-RPC, the trend of change in compressive strength was similar at all temperatures, i.e., the compressive strength increased as the content of recycled material enhanced from 0 to 50%, then the strength reduced at 75% replacement level. For RFA-NSC, a 6.31% increase in compressive strength was observed at 50% replacement with natural sand, while RFA-RPC exhibited a 12.57% increase in compressive strength at the same replacement level. Previous studies have also shown an increase in compressive strength after the replacement of natural sand with RFA. This increase was probably due to the internal curing effect of RFA, where initially absorbed water in the pores of recycled materials became available at a later age to promote further hydration process of cementitious materials [35,36,82]. The second reason was the filler effect, caused by the finer fraction of RFA, whose size was even smaller than natural sand, and subsequently, its greater specific surface area [45,83,84], resulted in filling the micro gaps of concrete making it denser and more compact, which helped to prevent early propagation of cracks. Additionally, the roughness and more irregular shape of RFA particles contribute to reinforce the transition zone of the recycled concrete, leading to a stronger bond with the cement paste [82,85]. Furthermore, non-hydrated particles present in both types of recycled materials played their role in the pozzolanic reaction [8,35,36,42]. As the percentage replacement of both the recycled materials increased to 75%, the specimens exhibited a decrease in strength at all temperature ranges. This decrease in strength was attributed to the excessive water present in the mix, which diminished the positive effect of RFA.

At any specific replacement ratio and temperature exposure, the compressive strength of samples containing RFA-RPC was higher than those containing RFA-NSC because RFA-RPC mixes contained RPC, which is included in the family of ultra-high performance concrete (UHPC) and the water-binder ratio was kept very low during its preparation. Due to this very low water-binder ratio, cement hydration in RPC usually falls in the range of 40–60% [8], making it possible for un-hydrated particles to participate in the hydration reaction when RFA-RPC is employed as a recycled material. On the other hand, RFA-NSC exhibited lower gain in compressive strength because RFA-NSC was originally produced with a relatively higher water-binder ratio, due to which, a lower number of non-hydrated cement particles was present for further hydration when used as a recycled material [86].

#### 4.4.2. Effect of Curing Temperature

The type of curing also affected the compressive strength behavior of the samples. The samples cured under hot water showed higher values of compressive strength as compared to those samples cured under normal water, at all replacement levels of fine aggregates and for all elevated temperature exposures. The gain in compressive strength for control samples was in the range of 6.74–9.76%. For RFA-NSC mixes the increase was in the range of 7.27–8.70% for 25% replacement, 5.37% to 7.29% at 50% replacement, and 3.92–19.39% at 75% replacement. For RFA-RPC mixes, the gain in compressive strength of hot cured samples was in the range of 5–8.86% at 25% replacement, 3.51–7.2% at 50% replacement, and 6.25–15.71% when 75% of natural sand was replaced with RFA-RPC. The increase in compressive strength due to the hot curing conditions is also reported by previous studies [32,33], which is due to the higher formation of calcium silicate hydrate in hot water curing through the pozzolanic reaction of silica fume and calcium hydroxide, more consumption of portlandite leading to the formation of a higher amount of hydration products [34], and secondary cementitious reactions at higher curing temperatures [34,35].

#### 4.4.3. Effect of PP Fiber Dosage

There was a slight reduction in the compressive strength of samples when the dosage of PP fibers increased from 0.5% to 1% at room temperature for both curing conditions, which was probably due to the inadequate mixing of PP fibers in the mix due to overdosage.

#### 4.4.4. Effect of Elevated Temperature

A decrease in compressive strength was observed when the samples under both types of curing conditions were subjected to elevated temperatures. It was due to a greater number of channels that were created after the melting of PP fibers at around 170 °C [87], which caused the excessive number of pores in the mix leading to strength reduction. For RFA-NSC mixes, the decrease was in the range of 3.6–11.7%, while for RFA-RPC mixes, a decrease of 2.3% to 9.7% was observed when the dosage of PP fibers was increased from 0.5% to 1%, at various temperature ranges. All samples cured under normal and hot water conditions exhibited a decrease in compressive strength when exposed to the elevated temperatures. The main reasons for the decrease in strength are: (1) decomposition and dehydration of hydration products; (2) coarsening of pore structures; and (3) thermal mismatch between aggregates and cement matrix [88]. Capillary and gel water evaporate at 100–150 °C, and chemically-bound water evaporates at 250–300 °C. Between 400 and 600 °C, calcium hydroxide dehydrates to calcium oxide. Loss of compressive strength at 600 °C was caused by the transformation of quartz from α-form to β-form [89] and also due to the coarsening of pore-structure of the hardened cement paste in concrete [90]. At this temperature, the matrix experiences volumetric expansion due to crystal phase transformation, causing dissolution of calcium silicate hydrate gel and calcium hydroxide, which further reduces the strength [91]. As shown in Figure 4 and Figure 5, the relative compressive strength of all samples indicates that the retention of compressive strength for samples cured in hot water was greater than that of the normal cured samples when subjected to the elevated temperatures. At 200 °C, the normal cured samples retained 87.4–93.77% of the compressive strength of controlled samples under similar curing conditions, while the hot cured samples retained 92.5–95.2% of the compressive strength of controlled samples under the hot water curing conditions. At 400 °C, the samples cured under normal conditions exhibited 70–82.8% of the compressive strength of the controlled samples, while the hot water cured samples retained 75.5–86.7% of the compressive strength of the controlled samples, under similar curing conditions. When the samples were tested after exposure to 600 °C, the samples cured under normal water exhibited the retention of 48.5–65% of controlled samples’ compressive strength, and the samples cured under hot water retained 57.8–70.1% of the compressive strength of controlled samples cured under similar conditions.

The experimental results were used to perform a regression analysis for the prediction of normalized compressive strength. The formulas for normalized compressive strength under normal and hot water curing conditions are given by Equation (1) with R^2^ = 0.917 and Equation (2) with R^2^ = 0.96, respectively:

(1) $$\frac{f_{cT}}{f_{c}}=1.005\;–\;2.8182\;\times \;10^{-4}\left(T\right)-\;6.43759\;\times \;10^{-7}\;\left(T^{2}\right)$$

(2) $$\frac{f_{cT}}{f_{c}}=1.00737-2.26141\;\times \;10^{-4}\left(T\right)-6.87916\;\times \;10^{-7}\;\left(T^{2}\right)$$

where $\frac{f_{cT}}{f_{c}}$ is the normalized compressive strength, $f_{cT}$ is the compressive strength at elevated temperature, $f_{c}$ is the compressive strength at room temperature, and T is the temperature, in the range of 25–600 °C.

### 4.5. Modulus of Elasticity

**Figure 8:**
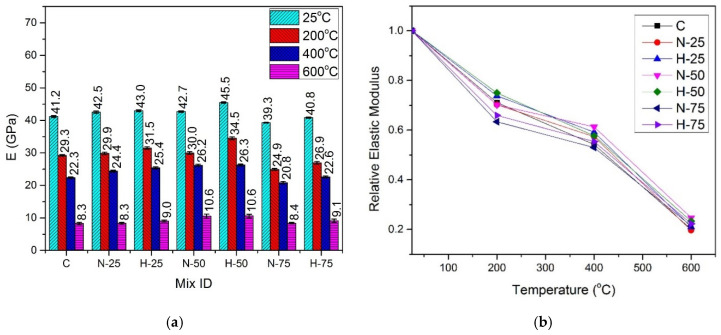
Elastic modulus of normal cured specimens (**a**) absolute, (**b**) relative.

**Figure 9:**
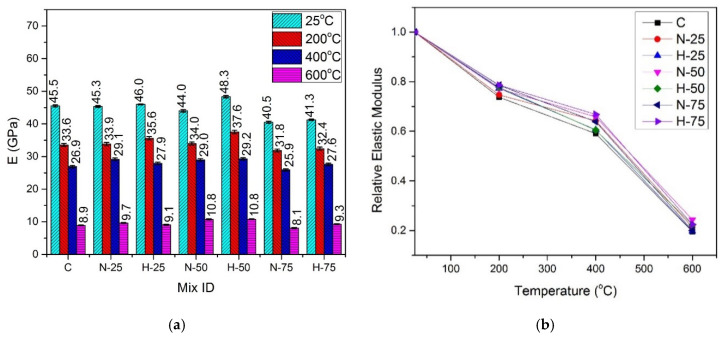
Elastic modulus of hot cured specimens: (**a**) absolute, and (**b**) relative.

Figure 8 and Figure 9 illustrate the values of absolute and relative elastic modulus for the samples subjected to the elevated temperatures and cured under normal water and hot water conditions, respectively.

#### 4.5.1. Effect of Recycled Aggregate Content and Type

The trend of change in the elastic modulus was similar to that of the compressive strength, split-tensile strength, and flexural strength, i.e., it increased with an increase in the amount of RFA-RPC and RFA-NSC till 50% replacement of sand, at all temperature exposures. At the 75% replacement level, the samples with both types of recycled materials exhibited a decrease in the elastic modulus under both curing conditions. At room temperature, under the normal curing conditions, the maximum elastic modulus value of 45.50 GPa was exhibited by the mix containing 50% RFA-RPC as compared to the control mix with 41.14 GPa. For the samples under hot curing conditions, the maximum elastic modulus for the same replacement level was 48.34 GPa, while it was 45.51 GPa for the control mix under similar curing conditions. At 75% replacement level, the elastic modulus decreased, exhibiting values of 39.30 GPa and 40.84 GPa for RFA-NSC and RFA-RPC mixes, respectively, under the normal curing conditions, while 40.52 GPa and 41.25 GPa for RFA-NSC and RFA-RPC mixes, respectively, under the hot curing conditions.

#### 4.5.2. Effect of Elevated Temperature and Curing Conditions

At 200 °C, the residual elastic modulus was in the range of 63.38–75.85% of the control mix for the normal cured samples and 73.75–78.62% of the hot cured control mix. At 400 °C, the normal cured samples retained 54.23–61.31% of elastic modulus while the residual elastic modulus of the hot cured samples was in the range of 59.09–66.84% of that of the control mix. There was a drastic decrease in the elastic modulus at 600 °C exposure, where the residual elastic modulus of the normal cured samples was in the range of 24.73–29.62% of that of the control mix under normal curing while it was in the range of 19.53–24.42% of that of the control mix under the hot water curing conditions. This drastic decrease can also be noticed from the results of mechanical properties. The reduction in elastic modulus is attributed to several factors such as chemical and physical changes, i.e., increased number of pores due to melted PP fibers, trapped vapor, decomposition of CH hydrates, C-S-H and Ca(OH)_2_, thermal incompatibility between aggregates, paste and steel fibers and cracking of the interfacial transition zone (ITZ) [91,92,93]. Modulus of elasticity of the hot cured samples at all temperature exposures was higher than that of the samples cured under normal water because of better pozzolanic reaction and, hence, denser growth of microstructure, which increased the stiffness of the hot cured samples [34,35,36].

Regression analysis was performed using the experimental data and the formula of elastic modulus under the normal curing condition is given by Equation (3) with R^2^ = 0.99, and formula for elastic modulus under the hot curing condition is given by Equation (4) with R^2^ = 0.99:

(3) $$\frac{E_{T}}{E}=1.0764\;-\;3.27212\;\times \;10^{-3}\left(T\right)+\;8.88761\;\times \;10^{-6}\;\left(T^{2}\right)-9.69846\;\times \;10^{-9}\;\left(T^{3}\right)$$

(4) $$\frac{E_{T}}{E}=1.05945\;-\;2.55474\;\times \;10^{-3}\left(T\right)+\;7.29025\;\times \;10^{-6}\;\left(T^{2}\right)-8.9683\;\times \;10^{-9}\;\left(T^{3}\right)$$

where $\frac{E_{T}}{E}$ is the normalized elastic modulus, $E_{T}$ is the elastic modulus at elevated temperature, *E* is the elastic modulus at room temperature, and T is the temperature in the range of 25–600 °C.

### 4.6. Peak Strain

**Figure 10:**
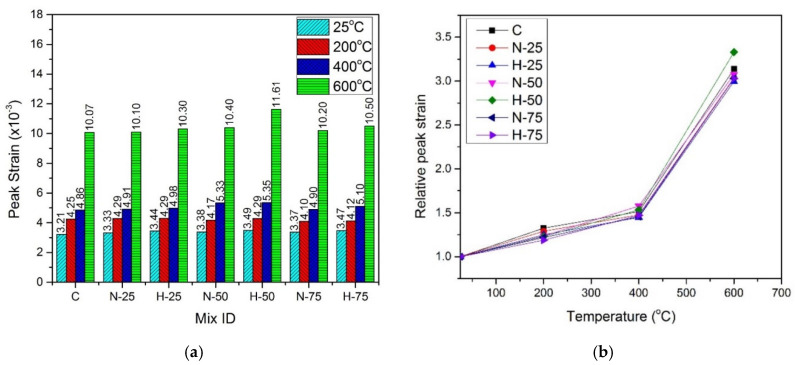
Peak strain of normal cured specimens (**a**) absolute, (**b**) relative.

**Figure 11:**
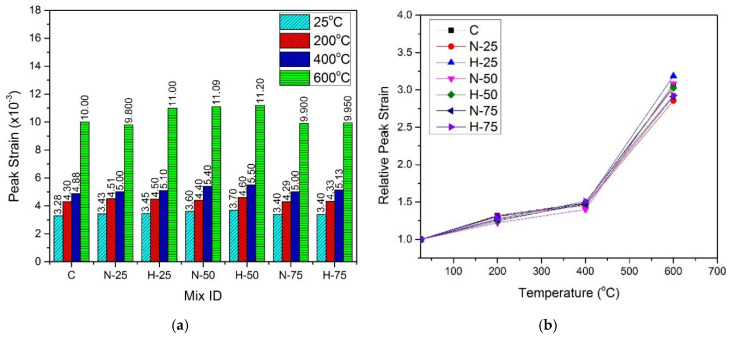
Peak strain of hot cured specimens: (**a**) absolute, and (**b**) relative.

Peak strain, corresponding to maximum stress, was recorded for all samples and is shown in Figure 10 and Figure 11 for normal cured and hot water cured samples, respectively. Peak strain exhibited an increasing trend with the increase in compressive strength at any specific temperature, due to the hot curing condition and replacement of sand with RFA till 50% replacement level. The type of RFA has a marginal effect on peak strain at any temperature. At room temperature, peak strain was in the range of 3.21 × 10^−3^ to 3.49 × 10^−3^, for the normal cured samples and in the range of 3.28 × 10^−3^ to 3.70 × 10^−3^ for the hot-cured samples. For any given sample, peak strain increased at elevated temperatures, under both curing conditions and for both types of recycled materials. At 200 °C, peak strain was in the range of 4.17 × 10^−3^ to 4.29 × 10^−3^ for the normal cured samples and 4.29 × 10^−3^ to 4.51 × 10^−3^ for the hot cured samples. Further increase in peak strain was observed for samples at 400 °C, where the normal cured samples and the hot cured samples exhibited a peak strain of 4.86 × 10^−3^ to 5.35 × 10^−3^ and 4.88 × 10^−3^ to 5.50 × 10^−3^, respectively. This increase was due to the cracks caused by the thermal incompatibilities between cement paste, ITZ, and steel fibers [94]. A more significant increase in peak strain was observed at 600 °C where the normal cured samples showed a peak strain in the range of 10.07 × 10^−3^ to 11.61 × 10^−3^ and the hot cured samples exhibited a peak strain in the range of 9.8 × 10^−3^ to 11.2 × 10^−3^. This significant increase in peak strain was due to the decomposition of hydration products, the transformation of the quartz phase, and the development of macro and micro cracks [95,96].

After performing regression analysis of experimental data, formula to predict normalized strain of samples cured under the normal curing conditions is given by Equation (5) with R^2^ = 0.99, and formula for samples cured under hot water is given by Equation (6) with R^2^ = 0.99:

(5) $$\frac{\varepsilon_{T}}{\varepsilon }=0.902291+\;4.37032\;\times \;10^{-3}\left(T\right)-\;1.9229\;\times \;10^{-5}\;\left(T^{2}\right)+3.00392\;\times \;10^{-8}\;\left(T^{3}\right)$$

(6) $$\frac{\varepsilon_{T}}{\varepsilon }=0.891558+\;4.83815\;\times \;10^{-3}\left(T\right)-\;2.07942\;\times \;10^{-5}\;\left(T^{2}\right)+3.10012\;\times \;10^{-8}\;\left(T^{3}\right)$$

where $\frac{\varepsilon_{T}}{\varepsilon }$ is the normalized strain, $\varepsilon_{T}$ is the peak strain at elevated temperature, ε is the peak strain at room temperature, and T is the temperature in the range of 25–600 °C.

### 4.7. Flexural Strength

**Figure 12:**
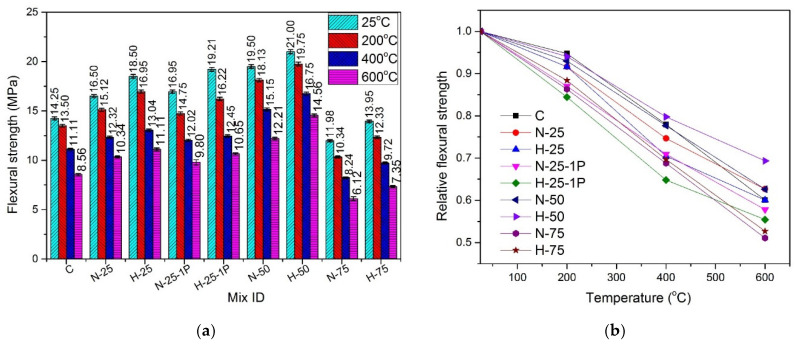
Flexural strength of normal cured specimens (**a**) absolute, (**b**) relative.

**Figure 13:**
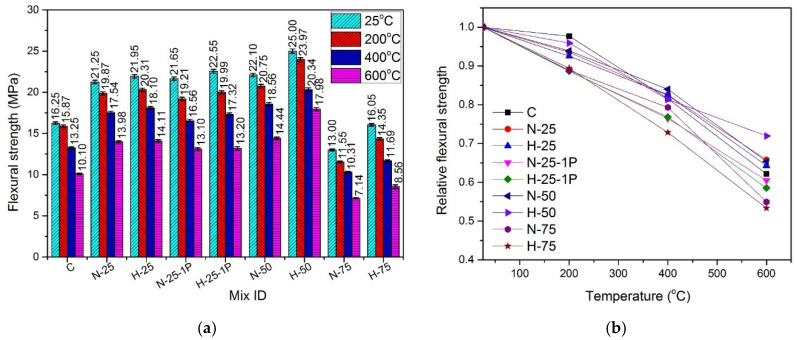
Flexural strength of hot cured specimens: (**a**) absolute, (**b**) relative.

The results of the flexural strength test for the specimens cured under normal water are shown in Figure 12 and the results of hot cured specimens are shown in Figure 13.

#### 4.7.1. Effect of Recycled Aggregate Content and Type

At any specific temperature, under both types of curing conditions, an increase in the flexural strength was observed when the quantity of RFA-NSC and RFA-RPC increased from 0% to 50%. At room temperature, as compared to controlled samples, this increase was in the range of 3.1–12.5% under the normal curing conditions and 1.5% to 6.7% for the hot cured samples. The gain in the flexural strength of samples containing RFA was attributed to better hydration reaction due to the presence of RFA, which caused the formation of denser C-S-H gel in the cement paste matrix, leading to a better bond between the matrix and the fibers [34,35,36]. The flexural strength of both RFA-RPC and RFA-NSC mixes decreased as the RFA content increased from 50% to 75% and this effect was more prominent in the samples containing RFA-NSC as compared to those containing RFA-RPC. The cause of this decrease at a higher volume of recycled aggregates was the presence of excessive water in the mix, which weakened the bond between recycled aggregates and cement paste [36,97,98].

#### 4.7.2. Effect of Hybrid Fibers

The use of hooked steel fibers improved the resistance against pullout while micro steel fibers and PP fibers formed a better network for bridging the cracks through widespread dispersion in the mix [10,11,12]. At room temperature, increasing the dose of PP fibers from 0.5% to 1% caused a slight increase in the flexural strength, i.e., 2.72% and 3.83% for RFA-NSC and RFA-RPC mixes, respectively, under the normal curing conditions, while in the case of hot water curing, the increase was 1.89% and 2.73% for RFA-NSC and RFA-RPC mixes, respectively. This increase was due to a greater number of fibers available in the mix for a better bridging of cracks.

#### 4.7.3. Effect of Elevated Temperature and Curing Conditions

A decrease in flexural strength was observed in all the samples as they were subjected to the elevated temperatures. The trend of decrease in strength for all samples was similar as the temperature increased from 25 to 600 °C. The presence of cracks induced by the elevated temperature reduces the effective area of cross-section and the presence of tensile stresses further widens those cracks. Due to this, the formation of microcracks at elevated temperature is considered more destructive for flexural strength as compared to compressive strength [99]. Under both curing conditions, samples with 50% recycled content exhibited higher strength retention as compared to other samples, while the sample with 75% recycled materials and those containing 1% PP fibers showed lower residual strength. The melting of PP fibers at around 170 °C causes the creation of more channels and thus makes the mix more porous, causing a rapid decrease in strength [91]. At 200 °C, residual flexural strength was in the range of 84.4–94.7% for the normal cured samples while for the samples cured in hot water, it was in the range of 88.6–97.6%. At 400 °C, it was in the range of 64.8–79.7% for the normal cured samples, and for the samples cured in hot water, it was in the range of 76.4–83.9%. For elevated temperatures, samples with 1% PP fibers exhibited lower flexural strength as compared to those containing 0.5% PP fibers, due to the creation of more channels and pores after melting of fibers [91].

The hot cured samples exhibited higher flexural strength at all temperature exposures, as compared to those cured in normal water. When the samples were subjected to the elevated temperatures, the rate of decrease of flexural strength in the hot cured samples was lower than that of the normal cured samples, due to better and denser microstructures caused by the hot water curing [12,13,32,33,34] leading to better resistance at elevated temperatures.

Regression analysis was performed using experimental data and the formula to predict flexural strength of the samples cured under normal conditions is given by Equation (7) with R^2^ = 0.93, and formula for the samples cured under hot water is given by Equation (8) with R^2^ = 0.93:

(7) $$\frac{f^{f}{}_{T}}{f^{f}}=1.03327-\;7.18237\;\times \;10^{-4}\left(T\right)$$

(8) $$\frac{f^{f}{}_{T}}{f^{f}}=1.00611-\;2.39948\;\times \;10^{-4}\left(T\right)-6.57091\;\times \;10^{-7}\left(T^{2}\right)$$

where $\frac{f^{f}{}_{T}}{f^{f}}$ is the normalized flexural strength, $f^{f}{}_{T}$ is the flexural strength at elevated temperature and $f^{f}$ is the flexural strength at room temperature, and T is the temperature in the range of 25–600 °C.

### 4.8. Split Tensile Strength

**Figure 14:**
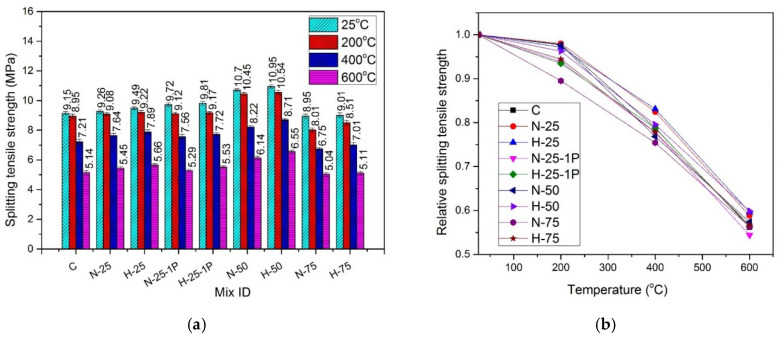
Split-tensile strength of normal cured specimens: (**a**) absolute, and (**b**) relative.

**Figure 15:**
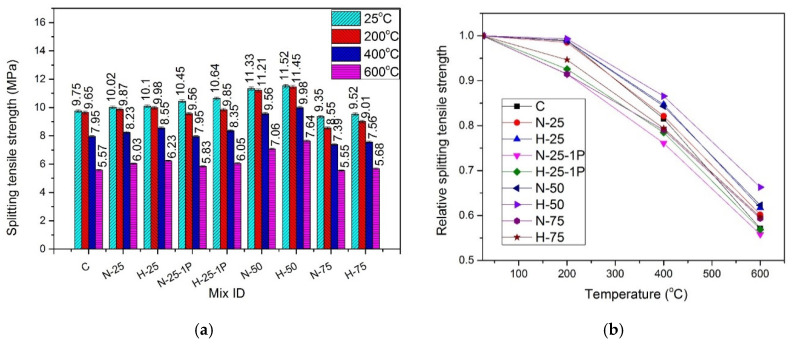
Split-tensile strength of hot cured specimens: (**a**) absolute, and (**b**) relative.

Figure 14 shows the split-tensile strength results of samples cured under the normal curing conditions and Figure 15 shows the split-tensile strength results of hot cured samples, exposed to various temperatures. The effect of amount and type of recycled materials, PP fiber dosage, and elevated temperature on the split-tensile strength was similar to that on compressive and flexural strengths, as previously discussed.

#### 4.8.1. Effect of Recycled Aggregate Content and Type

At all temperature exposures, the split-tensile strength kept on increasing until 50% sand replacement with RFA-NSC and RFA-RPC, under both curing conditions. This increase was associated with the improvement in the bond between the hybrid fibers and denser hydration products that were formed during the hydration reaction of un-hydrated recycled material [8,35,36,42]. At 75% replacement, a decrease in split-tensile strength was observed. At room temperature, under the normal curing conditions, split-tensile strength increased in the range of 1.28–16.95% for the samples with RFA-NSC, while the samples containing RFA-RPC showed an increase in the range of 3.72–19.67% of the strength of the control mix. The samples under hot water curing exhibited an increase in the range of 2.77–16.28% when RFA-NSC was used and the increase was in the range of 3.54–18.24% of the strength of the control mix when RFA-RPC was added as a sand replacement under same curing condition. At any temperature exposure and RFA replacement level, samples containing RFA-RPC showed higher strength as compared to those containing RFA-NSC, due to the reasons discussed in Section 4.4.1.

#### 4.8.2. Effect of Elevated Temperature and Curing Conditions

When the samples were exposed to elevated temperatures, the split-tensile strength started decreasing. At 200 °C, the normal cured samples retained 89.4–97.9% of split-tensile strength while the residual strength of the hot cured samples was in the range of 91.4–99.3%. At 400 °C, residual split-tensile strength for the normal cured samples was in the range of 75.4–83.1% and for the hot cured samples, it was in the range of 79.03–86.5%. At 600 °C, the relative strength was in the range of 54.4–59.6% for the normal cured samples and 55.77–66.2% for the hot cured samples. A decrease in split-tensile strength at the elevated temperatures was mainly due to the decomposition of hydration products, which weakens the bond between fibers and the hydration products [91].

The hot cured samples displayed higher values of split-tensile strength at all temperature exposures, as compared to those cured under normal water. A similar effect of the hot water curing was observed in other mechanical properties tests.

#### 4.8.3. Effect of Fibers

At room temperature, an increase in strength was observed when PP fibers dosage was increased from 0.5% to 1%. The increase was 4.11% for RFA-NSC mix and 5.11% for RFA-RPC mix. This increase in strength may be attributed to the higher resistance offered by a greater number of fibers available against the tensile stresses, which helps to prevent crack propagation [100].

The addition of hybrid fibers played an important role in avoiding the brittle failure of the samples at room and elevated temperatures by keeping them intact and preventing the sudden explosive failure of RPC. The use of hooked fibers prevented the pullout of concrete, while micro straight fibers prevented the propagation cracks through widespread and uniform dispersion in the matrix. PP fibers play an important role in preventing spalling of the samples at elevated temperatures since they have a melting point at around 167 °C. The fibers melt after reaching this temperature, and provide escape channels for trapped vapors and reduce the vapor pressure [59]. Increasing the dosage of PP fibers to 1% exhibited a negative effect at elevated temperatures due to a larger number of pores created after the melting of a greater number of PP fibers, leading to an earlier failure as compared to the samples containing 0.5% PP fibers [71].

Regression analysis was carried out using experimental data to predict the values of split-tensile strength under both curing conditions. Equation (9) gives the formula for the normal cured specimens with R^2^ = 0.98, and Equation (10) gives the formula for the hot cured specimens with R^2^ = 0.97.

(9) $$\frac{f^{s}{}_{T}}{f^{s}}=1.00741\;–\;9.59872\;\times \;10^{-5}\left(T\right)-\;1.04178\;\times \;10^{-6}\;\left(T^{2}\right)$$

(10) $$\frac{f^{s}{}_{T}}{f^{s}}=1.00254+\;4.39461\;\times \;10^{-5}\left(T\right)-\;1.17194\;\times \;10^{-6}\;\left(T^{2}\right)$$

where $\frac{f^{s}{}_{T}}{f^{s}}$ is the normalized split-tensile strength, $f^{s}{}_{T}$ is the split-tensile strength at elevated temperature, $f^{s}$ is the split-tensile strength at room temperature, and T is the temperature in the range of 25–600 °C.

### 4.9. Water Absorption

**Figure 16:**
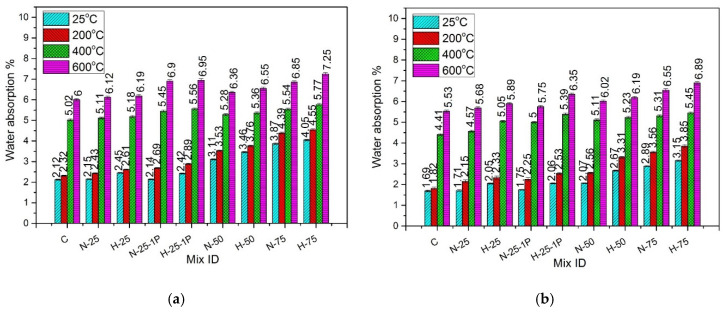
Percentage water absorption of specimens: (**a**) normal cured, and (**b**) hot cured.

Figure 16 presents the percentage water absorption of the samples exposed to normal and hot water curing and subjected to various temperatures.

#### 4.9.1. Effect of Recycled Aggregates Content and Type

At all temperature exposures, an increase in recycled aggregate content from 0% to 75% caused an increase in the percentage water absorption. The higher water absorption capacity of RFA contributed towards the increase in the percentage water absorption of RPC mixes [101,102]. Moreover, as reported in the previous researches, particle size gradation of RFA was also responsible for the higher water absorption. As compared to natural sand, part of RFA contains larger particles, which cause connectivity between the grains, leading to increased capillary absorption capacity and percentage water absorption with an increase in RFA content [37,103,104,105]. Therefore, the samples containing RFA-RPC exhibited higher water absorption as compared to those containing RFA-NSC due to the higher water absorption capacity of RFA-RPC.

#### 4.9.2. Effect of Elevated Temperature and Curing Conditions

The percentage water absorption showed a rising trend as the exposure temperature increased from 25 °C to 600 °C. For the normal cured samples, the increase was in the range of 8.62–20.45% at 200 °C, 29.81–60.73% at 400 °C, and 43.5–68.9% at 600 °C. For the hot cured samples, the increase in the percentage water absorption was in the range of 7.17–22.12% at 200 °C, 42.2–65% at 400 °C, and 54.28–69.89% at 600 °C. The increase in percentage water absorption at 200 °C was attributed to the penetration of water in channels created due to the melting of PP fibers [91], whose melting point is 167 °C. Beyond 200 °C, a rise in percentage water absorption was due to the decomposition of hydration products, which led to increased porosity in the internal matrix [71].

All the hot cured samples, containing either RFA-NSC or RFA-RPC, exhibited lower water absorption as compared to those cured in normal water. This decreased water absorption due to hot curing can be attributed to the better pozzolanic reaction resulting in a denser microstructure, which eventually contributes to filling the voids [34]. This trend was observed for the samples at all temperature exposures.

#### 4.9.3. Effect of PP Fiber Dosage

At room temperature, samples with 1% PP fibers showed no significant change in the percentage water absorption as compared to those containing 0.5% PP fibers. But at the elevated temperatures, specimens with 1% PP fibers exhibited higher water absorption than those containing 0.5% PP fibers. Samples with 1% PP fibers exhibited an increase in water absorption in the range of 4.69–10.69% and 6.78–10.72% for RFA-NSC and RFA-RPC mixes, respectively, as compared to those containing 0.5% PP fibers, under the normal curing conditions. For the samples cured under hot water, the increase was in the range of 5.21–11.79% for RFA-NSC mixes and 6.53–10.69% for RFA-RPC mixes. This increase was due to a greater number of channels created due to the melting of the high dosage of PP fibers at elevated temperatures [71].

## 5. Conclusions

In this study, RPC was prepared using two different types of recycled fine aggregates (RFA) and the elevated temperature performance was evaluated. Various properties including compressive strength, stress-strain curves, elastic modulus, peak strains, split-tensile strength, flexural strength, and water absorption were studied at 25, 200, 400, and 600 °C. From the experimental results, the following conclusions can be drawn:

1. No spalling was found in any of the RPC samples when subjected to elevated temperatures. PP fibers helped to prevent the explosive spalling of the specimens.
2. The flow of RPC reduced significantly due to an increase in the dosage of PP fibers. However, the flow gradually increased with an increase in RFA content.
3. There was no significant improvement in the mechanical properties of RPC mixes when PP fiber dosage was increased from 0.5% to 1% by volume of RPC at room temperature. At the elevated temperatures, mixes with 0.5% PP fibers exhibited better performance than those containing 1% PP fibers.
4. The compressive strength, split-tensile strength, and flexural strength improved at all temperature exposures as the RFA content increased from 0% to 50%. The maximum increase in strength was observed at 50% replacement of sand with RFA.
5. The specimens containing RFA-RPC exhibited better performance at all temperature exposures against compressive strength, split-tensile strength, and flexural strength tests, as compared to those containing RFA-NSC.
6. Water absorption of the samples increased with an increase in the RFA content and the exposure temperature. Specimens containing RFA-RPC exhibited higher water absorption values as compared to those containing RFA-NSC. Specimens with 1% PP fibers exhibited higher water absorption as compared to those with 0.5% PP fibers.
7. The compressive stress-strain response of the specimens indicated that the moduli of elasticity degraded gradually with an increase in temperature exposure up to 400 °C. A significant decrease in moduli of elasticity was observed at 600 °C. At any specific temperature, an increase in RFA content had a positive effect on moduli of elasticity up to 50% replacement level. Peak strain of all RPC mixes increased as the temperature increased from 25 to 600 °C.
8. The specimens cured under hot water exhibited better performance as compared to those cured under the normal curing conditions due to better hydration reactions at a higher temperature. This behavior was observed for all the specimens at room temperature as well as at elevated temperatures.
9. RFA can be used as a partial replacement of natural sand to produce eco-friendly and sustainable RPC that can be helpful to address the problems of waste disposal and can also assist in preventing the over mining of river sand.

## 6. Recommendations for Further Research

The scope of current research work was limited to the study of some properties of RPC containing two types of RFA at room and elevated temperature. Future research is needed to fully understand the behavior of RPC under various following conditions:

- The effect of moisture content and particle gradation of RFA, and the effect of other dosages and length of PP fibers on the spalling behavior of RPC containing RFA need to be studied.
- The durability study of RPC containing RFA, such as corrosion resistance and chloride permeability may also be conducted. It will give further insight into the long-term behavior of this type of RPC.
- The microstructural analysis of this type of RPC will be beneficial to understand its behavior in more detail.
- The study of the creep behavior of this RPC will give a clear picture of long-term performance under an applied load.
- The effect of member size on the behavior of this RPC at elevated temperatures may also be studied, which will give a more accurate prediction of the performance of full-scale RPC frames under fire.

Thus, the current and future studies on this topic will help the construction industry to utilize RFA in the production of sustainable RPC with more confidence.

## Figures and Tables

**Table 1:** Properties of binders.

Compounds%	Cement	Silica Fume
Chemical Composition		
SiO_2_	21.50	93.45
Al_2_O_3_	05.25	00.89
Fe_2_O_3_	03.25	01.98
CaO	62.50	00.47
MgO	02.50	00.95
SO_3_	02.95	00.33
Na_2_O	00.25	00.42
K_2_O	01.25	01.30
Loss on ignition	0.75	-

**Table 2:** Physical properties of binders.

Physical Properties	Cement	Silica Fume
Specific surface area (m^2^/kg)	309.0	14,000
Specific gravity	3.03	2.17
Initial setting time (min)	55	-

**Table 3:** Physical properties of the materials used as fine aggregates.

Aggregates Type	Fineness Modulus	Bulk Density (kg/m^3^)	Water Absorption (%)
Natural sand	2.39	1503	0.91
RFA-NSC	2.94	1454	5.26
RFA-RPC	2.91	1488	6.76

**Table 4:** Properties of steel fibers.

Type		Dia. (mm)	Length (mm)	Tensile Strength (MPa)	Density (g/cm^3^)
Steel fibers	Straight	0.2 ± 0.03	13 ± 1	2850	7.81
	Hooked	0.2 ± 0.03	22 ± 1	2850	7.81
PP fibers	Straight	0.0445	6.20	308	0.946

**Table 5:** Mix proportion of various concrete mixtures (relative mass ratio with respect to cement).

Mix ID	Free W/B	RFA%	Cement	Silica-Fume	Sand	RFA-NSC	RFA-RPC	SP *	Fibers **
C	0.21	0	1	0.25	1.1	0	0	2	1%H + 1%S + 0.5%PP
N-25	0.21	25	1	0.25	0.825	0.275	0	2	1%H + 1%S + 0.5%PP
H-25	0.21	25	1	0.25	0.825	0	0.275	2	1%H + 1%S + 0.5%PP
N-25-P1	0.21	25	1	0.25	0.825	0.275	0	2	1%H + 1%S + 1%PP
H-25-P1	0.21	25	1	0.25	0.825	0	0.275	2	1%H + 1%S + 1%PP
N-50	0.21	50	1	0.25	0.550	0.550	0	2	1%H + 1%S + 0.5%PP
H-50	0.21	50	1	0.25	0.550	0	0.550	2	1%H + 1%S + 0.5%PP
N-75	0.21	75	1	0.25	0.275	0.825	0	2	1%H + 1%S + 0.5%PP
H-75	0.21	75	1	0.25	0.275	0	0.825	2	1%H + 1%S + 0.5%PP

* % by mass of the binder, ** % by volume of concrete (H = hooked steel fibers, S = straight steel fibers, PP = polypropylene fibers).

**Table 6:** Overview of test methods and specimens.

Tests	Standard	Specimen Type	Dimension (mm)
Compressive strength	ASTM C109 [74]	Cubes	50 mm × 50 mm × 50 mm
Split-tensile strength	ASTM C496 [75]	Cylinders	100 mm × 200 mm
Flexural strength	ASTM C293 [76]	Prisms	40 mm × 40 mm × 160 mm
Water Absorption	ASTM C642 [77]	Cubes	50 mm × 50 mm × 50 mm
